# The Prevalence of Double Eyelid and the 3D Measurement of Orbital Soft Tissue in Malays and Chinese

**DOI:** 10.1038/s41598-017-14829-4

**Published:** 2017-11-01

**Authors:** Ting Yin Lu, Kathreena Kadir, Wei Cheong Ngeow, Siti Adibah Othman

**Affiliations:** 1 Department of Oral and Maxillofacial Clinical Sciences, Faculty of Dentistry, University of Malaya, 50603 Kuala Lumpur, Malaysia; 2 Department of Paediatric Dentistry and Orthodontics, Faculty of Dentistry, University of Malaya, 50603 Kuala Lumpur, Malaysia

## Abstract

This study aimed to determine the prevalence of double eyelid among two main Mongoloid ethnicities, the Malays and Chinese who reside in Malaysia. We also measured their periorbital tissue parameters for application in anthropology, optometry, ophthalmology, oculoplastic surgery and maxillofacial trauma surgery. The images of the 103 Malay and 97 Chinese volunteers were captured using indirect 3D photogrammetry, and quantitative measurement was obtained using the software provided by the manufacturer. All Malays and 70.1% of Chinese in this cross section population had double eyelid on both eyes. The mean pretarsal skin height was 3.99 mm for the Malays and 2.29 mm for the Chinese. The Malays appeared to have shorter eyebrow height (11.10 mm) compared to the Chinese (11.79 mm). An opposite pattern could be seen in the measurement of upper eyelid crease height between the Malays (8.33 mm) and the Chinese (4.91 mm). Of note, the intercanthal distance of the Chinese (IDC = 35.85 mm) was wider and their interpupillary distance was narrower (IPD = 62.85 mm) compared to the Malays’ (ICD = 34.21 mm; IPD = 64.04 mm). In conclusion, there were significant differences in the prevalence of double eyelid and periorbital tissue measurements between the Malays and Chinese.

## Introduction

The eye, upper eyelids and eyebrow are the most prominent anatomy structures in the middle third of the face and can serve as distinctive features for anthropology and forensic studies. Some important features associated with these anatomy structures are the colour of the pupil, the size of the periorbital soft tissues, and the presence or absence of an anti-mongoloid slant or double eyelid. The first and last features have been used by some ethnic groups to determine beauty and facial aesthetic^1^. However, these orbital features vary between different ethnic groups and even among individuals within the same ethnic group.

Double eyelid is the layman’s term used to describe the presence of an upper eyelid crease, which is also known as the supratarsal fold or palpebral fold^2^. It appears due to terminal interdigitation of levator aponeurosis inserting into the subdermal surface of upper lid skin. Currently, it has been expected that approximately half of the Asian populations do not have an upper eyelid crease, however, details of the ethnic groups involved is incomplete^3^.

There are only several studies that have been conducted to determine the prevalence of double eyelid among Asians, as summarised in Table 1 ^2–8^. The earliest literature on the prevalence of double eyelids in Asian was recorded by Mikamo^2^ who reported the first case of Asian blepharoplasty on Japanese women, mentioning that 17 to 18% of his Japanese female patients did not have upper eyelid crease. The prevalence of upper eyelid crease (or *double eyelid*; this term will be used thereafter in this report) among Chinese female was reported at between 66.7% and 83.1%, which is close that reported by Mikamo^2,4,5^. The prevalence of double eyelids among Koreans appears to be lower than the Japanese and Chinese (Table 1). The prevalence of double eyelids in other Asian ethnicities is unknown.

**Table 1:** Key studies that reported the prevalence of double eyelid among Asian populations.

Authors	Year	Ethnics	Prevalence of double eyelid (%)
Mikamo^2^	1896	Japanese	82–83 (female)
Sim, Smith & Chan^4^	2000	Singapore Chinese	66.7 (female)
Song *et al*.^6^	2007	Korean	24.1 (male) 45.5 (female)
Park *et al*.^7^	2008	Asian	30.3 (male) 41.3 (female)
Nguyen, Hsu & Dinh^3^	2009	Asian	50.0
Chen, Ma, & Liao^5^	2013	Taiwanese Chinese	83.1 (female)

A literature search showed that only a few studies had described the soft tissue parameters of the eyes and orbits of the Malay and Chinese ethnic groups. Both Malays and Chinese are Asian of Mongoloid stock and are two of the largest ethnic groups in Malaysia, a multi-racial country in South East Asia. The Malays and Chinese make up of 50.1% and 22.6% of the population of Malaysia, respectively^9^. The pretarsal skin height (PTSH) among the Malays was reported to range from 2.9 mm to 3.0 mm by Dharap & Reddy^10^ while that for the Chinese was less, at 2.4 mm to 2.6 mm, as reported by Chen *et al*.^5^. In terms of eyebrow height (EH), Dharap & Reddy^10^ reported that the Malays had an eyebrow height of between 11.0 mm and 11.7 mm, while Wu *et al*.^11^ reported that the Chinese had a higher eyebrow height of between 12.11 mm and 12.53 mm. The upper eyelid crease height (UECH) of the Malays ranged from 6.1 mm to 6.6 mm^10,12^; in comparison, similar parameter in the Chinese ranged from 4.0 mm to 6.1 mm^4,5,12^. Table 2 summarises the available key findings on the periorbital tissues of Asians and compare them with the Caucasian and African American. In all, it can be seen that these studies show that wide variations happen in all 3 parameters, even within the same ethnic group.

**Table 2:** A summary of key studies on the pretarsal skin height, eyebrow height and upper eyelid crease height of Asians in comparison to Caucasian and African American.

Authors	Ethnic	Pretarsal Skin Height (mm)	Eyebrow Height (mm)	Upper Eyelid Crease Height (mm)
Cartwright *et al*.^20^	Caucasian	1.6 (M) 2.8 (F)	6.4 (M) 7.6 (F)	6.9 (M) 7.3 (F)
Dharap & Reddy^10^	Malay	2.9 (M) 3.0 (F)	11.7 (M) 11.0 (F)	6.1 (M) 6.5 (F)
Sim, Smith & Chan^4^	Singapore Chinese	—	—	6.0 (F)
Park *et al*.^7^	Asian	—	12.4 (M) 12.0 (F)	6.6 (M) 6.5 (F)
Price *et al*.^26^	African American	3.1 (M) 3.6 (F)	18.9 (M) 21.7 (F)	7.2 (M) 7.7 (F)
	Caucasian	2.0 (M) 3.3 (F)	14.1 (M) 20.7 (F)	6.2 (M) 7.5 (F)
Wu *et al*.^11^	Chinese	—	Right	—
			12.11 (M)12.53	
			(F)Left	
			12.22 (M) 12.50 (F)	
Preechawai^12^	Chinese	—	—	4.0
	Malay	—	—	6.6
Chen, Ma & Liao^5^	Chinese	2.4* 2.6^¶^	—	6.0* 6.1^¶^

M = Male, F = Female; *21–30 years old, ^¶^31–40 years old.

Aljunid & Ngeow^13^ conducted the only manual anthropometric study to determine the intercanthal and interpupillary distances of the Malays and Chinese in Malaysia. They concluded that Chinese’s intercanthal distance (ICD) was significantly wider than Malays’. On the contrary, Aljunid & Ngeow^13^ reported no significance difference in interpupillary distance (IPD) between their Malay and Chinese subjects. The reported ICD among the Chinese was from 34.7 mm to 40.01 mm^11,13–17^, and the IPD among the Chinese was from 61.4 mm to 64.8 mm^13^. In general, based on the soft tissue measurements and intercanthal distance, the Malay appears to have larger eyes that are located closer together.

Asians born with double eyelid appear to have a wider palpebral fissure with larger eyes and are culturally perceived to be more pleasing and friendly than those without double eyelid^1^. Moreover, double eyelid allows women greater room for applying cosmetic on the upper eyelid. While a single eyelid can be converted to become double eyelid, the same cannot be true for the soft tissue parameters, which is what one is born with. Therefore it is not difficult to understand that the demand to surgically create double eyelid is high among people without such an appearance, as having double eyelid will most probably make them more attractive. Such surgery is termed blepharoplasty. There is currently an upward trend in Asian Blepharoplasty among East Asian populations comprising the Chinese, Korean and Japanese^18^.

Nevertheless, there is a void in the literature regarding the detailed feature, prevalence of double eyelid and the measurements of the orbital parameters among the Malays, except for some incomplete descriptions in several literatures^10,12,13^. Personal experiences of the authors perceive that the Malay show distinctive facial features when compared to the Chinese, especially in the periorbital region. One of the distinctive features is the lack of double eyelid among Chinese, while the Malays are perceived as not having such issue. A study in the neighboring country, Singapore, reported a prevalence of 66.7% for the presence of double eyelid among a selected group of female Chinese subjects examined^4^. Having said so, there are occasions where members of one ethnic group are mistaken for the other, leading to some social and cultural embarrassments.

Until now, to our knowledge there is no published data that compare the prevalence of double eyelid and periorbital parameters between the Malays and Chinese, obtained using 3D photogrammetry. Therefore, this study was performed using non-invasive 3D photogrammetry to determine the prevalence of double eyelid of these 2 ethnic groups, and to measure the periorbital tissue which will also help to determine if both eyes of the same subject were symmetry.

## Results

The subjects of this study consisted of a convenient sample of 200 patients (103 Malay and 97 Chinese) aged between 20 and 39 years old with the additional inclusion criteria of the Malay or Chinese ethnics having no history of mixed parentage for the past 3 generations. Of the 103 Malay subjects, 46 were males and 57 were females, while 49 males and 48 females made up the 97 Chinese subjects recruited. The mean age of the sample population was 25.62 years with standard deviation of 4.26 years.

No statistical significance was found between manual anthropometry and 3D anthropometry in all the eight parameters measured (*p* > 0.05; Table 3). All ICC indices were found to be in good agreement (0.868–0.977), indicating that the data obtained using 3D photogrammetry was reproducible as those obtained using the conventional manual measurement technique. Additional analysis done using paired sample *t*-test confirmed that there were no differences in the measurements obtained using these 2 different techniques. In summary, the reliability of data collected using VECTRA-3D 360 5 pod-mounted photosystem (Canfield Scientific Inc., Fairfield, NJ, USA) was considered high.

**Table 3:** Determination of the reliability of 3D photogrammetry.

Parameters	Methods of measurements (mm)		Paired Sample *t*-test	Interclass Correlation Coefficient
	Manual	3D photogrammetry		
ICD	35.45	35.59	0.970	0.977
IPD	63.70	64.52	0.810	0.868
PTSH (R)	3.65	3.54	0.870	0.925
PTSH (L)	3.85	4.25	0.881	0.938
EH (R)	9.15	9.31	0.817	0.893
EH (L)	9.85	10.25	0.817	0.920
UECH (R)	8.55	8.92	0.874	0.918
UECH (L)	8.95	9.33	0.893	0.923

Analysis of the first and second set of 10% of the collected data showed a Cronbach’s alpha of 1. The Bland and Altmann test that was used to determine the error of intra-examiner calibration found only 0.87% differences in measurement, suggesting a very high reliability and reproducibility. This finding was further supported by paired sample *t*-test (*P* = 0.442) and intraclass corelation coefficient (*P* < 0.001).

In summary it was discovered that all Malay subjects regardless of gender were found to have double eyelid on both eyes. However, among the Chinese, the prevalence of double eyelid on both eyes involved slightly more than two-third (70.1%) of subjects. The prevalence of double eyelid among Chinese female at 81.3% was higher than that observed for the male (59.2%). However no association was found between genders with the prevalence of double eyelid of the Chinese subjects (Chi-square test; *P* > 0.05).

Table 4 shows the measurements of PTSH, EH and UELCH of the Malays was 3.99 mm, 11.10 mm and 8.33 mm, respectively. In contrast similar parameters in the Chinese measured at 2.29 mm, 11.79 mm and 4.91 mm respectively. It was observed that all periorbital measurements showed statistical significance between the Malays and Chinese. The Malays had higher PTSH (p < 0.05) and UELCH (p < 0.05) compared to the Chinese. In fact, the PTSH and UELCH were several millimeters higher in the Malays. However, the Chinese presented with a higher EH (p < 0.05). There was no gender difference in all measurement except for the UELCH between Chinese male and female, where it was significantly higher in male (Table 4).

**Table 4:** Measurements of PTSH, EH and UELCH among Malay and Chinese.

Parameters	**Malay**	**Chinese**
	Mean ± SD (95% Confidence Interval) mm	
**PTSH**		
Male	3.93 ± 1.47 (3.49–4.36)	1.91 ± 1.99 (1.34–2.48)
Female	4.04 ± 1.10 (3.75–4.34)	2.68 ± 1.62 (2.21–3.15)
Average^**#**^	3.99 ± 1.28 (3.74–4.23)	2.29 ± 1.85 (1.92–2.67)
**EH**		
Male	11.42 ± 2.01 (10.82–12.01)	11.67 ± 2.32 (11.00–12.33)
Female	10.84 ± 1.90 (10.33–11.34)	11.93 ± 2.08 (11.32–12.53)
Average*	11.10 ± 1.97 (10.71–11.48)	11.79 ± 2.20 (11.35–12.24)
UELCH*		
Male	8.05 ± 2.11 (7.42–8.67)	3.95 ± 3.57 (2.93–4.97)**
Female	8.56 ± 1.80 (8.09–9.04)	5.89 ± 3.16(4.98–6.81)**
Average^**#**^	8.33 ± 1.95 (7.95–8.71)	4.91 ± 3.49 (4.21–5.61)

^*^Independent t-test; *p* < 0.05, **Independent t-test; *p* < 0.05.

^#^Mann-Whitney test; p < 0.05.

PTSH, pretarsal skin height; EH, eyebrow height; UELCH, upper eyelid crease height.

The presented values for PTSH, EH and UECH are the average value for the right and left side. Further details that compare between sides are shown in the asymmetry between right and left eyes’ periorbital measurements below.

Table 5 shows the intercanthal and interpupillary distances of the Malays and Chinese. The Malays had an ICD of 34.21 ± 2.74 mm while the IPD was 64.04 ± 3.31 mm. In comparison, the ICD of the Chinese was 35.85 ± 2.47 mm, while their IPD was 62.85 ± 3.08 mm. There were statistically significant differences in intercanthal and interpupillary distances between the Malays and Chinese, with the ICD for the Chinese being wider than the Malays’ (p < 0.05), but the latter’s IPD was wider than the Chinese’s (p < 0.05). Within the same ethnic group, it was discovered that the ICD and IPD among male were significantly wider than female (p < 0.05).

**Table 5:** Measurements of intercanthal and interpupillary distances among the Malay and Chinese.

Parameters	Malay	Chinese
	Mean ± SD (95% Confidence Interval) mm	
**ICD**		
Male	34.91 ± 2.95 (34.04–35.79)	36.36 ± 2.46 (35.65–37.06)
Female	33.64 ± 2.43 (33.99–34.28)	35.34 ± 2.40 (34.65–36.04)
Average*	34.21 ± 2.74 (33.67–34.74)	35.85 ± 2.47 (35.36–36.35)
**IPD**		
Male	66.03 ± 2.96 (65.15–66.91)	64.37 ± 2.59 (63.62–65.11)
Female	62.44 ± 2.66 (61.44–63.15)	61.31 ± 2.76 (60.50–62.11)
Average*	64.04 ± 3.31 (63.39–64.69)	62.85 ± 3.08 (62.23–63.47)

^*^Independent t-test; *p* < 0.05.

ICD = Intercanthal distance; IPD = Interpupillary distance.

In line with our last objective, Table 6 shows the measurement as well as the differences in PTSH, EH and UELCH between the right and the left eye. In summary, all measurements were significantly larger in the left eye when compared to the contralateral side (*p* < 0.05).

**Table 6:** Asymmetry between Right and Left Eyes’ Periorbital Measurements.

Parameters	Side	Measurements	Significance
Pretarsal Skin Height	Right	2.99 ± 1.77	<0.001
	Left	3.35 ± 1.81	
Eyebrow Height	Right	11.21 ± 2.13	<0.001
	Left	11.65 ± 2.08	
Upper Eyelid Crease Height	Right	6.42 ± 3.21	<0.001
	Left	6.92 ± 3.35	

## Discussion

The prevalence of double eyelid in Malaysian Chinese (70.1%) as observed in this current research is higher than expected of Asian population^3^. Sim *et al*.^4^ reported two third of the 100 Singaporean Chinese women (≈66.7%) in their study had double eyelid. The prevalence of double eyelid among Chinese female in the current research was 81.3%, which is higher than the prevalence reported by Sim *et al*.^4^ on Singaporean Chinese female, but is close to that reported by Chen *et al*.^5^ on Taiwanese Chinese female. A weighed Chi-Square test was conducted to compare the prevalence of double eyelid in Sim *et al*.’s^4^ study with our study, however the difference was found to be not statistically significant. The prevalence of double eyelid from our study was within those reported by these two previous studies, indicating a lot of similarity exits between Chinese populations from Malaysia, Singapore and Taiwan who share the same cultural and perhaps ancestral origin.

In the current study, the prevalence of double eyelid among the Malays was 100%. Even though Dharap & Reddy^10^ measured the upper eyelid crease height in the Malays, the prevalence of double eyelid among their Malay subjects was not mentioned. The current finding confirms the authors’ intuition that single eyelid is not common in the Malay ethnic. Thus this result would be able to fill the void in the literature, where the prevalence of double eyelid among the Malays has not been reported previously.

The finding of our study hints that the absence of upper eyelid crease in Malaysian population is more likely to occur in the Chinese ethnic group. However, the presence of double eyelid does not necessary conclude any specific ethnicity unless further study on the location and shape of the eyelid crease is done. For example Preechawai^12^ mentioned that the Thai-Malay ethnic had significant parallel lid crease. Further study on the characteristics and features of double eyelid is necessary to elicit these information.

Dharap & Reddy^10^ conducted a periorbital study on the Malay subjects in Malaysia more than 2 decades ago. In their study, the mean pretarsal skin height for male and female was reported as 2.9 mm and 3.0 mm respectively. In this current study, the mean pretarsal skin height for Malay male was 3.93 mm and for Malay female was 4.04 mm. The result obtained from 3D photogrammetry was higher compared to manual anthropometry utilized by Dharap & Reddy^10^. This lower reading may result from possible upper eyelid movement when the measuring ruler was place near to the subjects’ eye. This movement can be ruled out in 3D photogrammetry as the subjects had to sit still with their Frankfurt plane held parallel to the floor and eyes placed in primary gaze. Having said so, our calibration study showed that measurements obtained using 3D photogrammetry did not differ much from the results obtained manually. Hence, it is possible that the size of the people in this ethnic group has increased throughout the last 20 years due to the availability of better diet and healthcare, hence the difference in the results obtained.

The result for eyebrow height of the Malays from current study did not differ much from that reportedly by Dharap & Reddy^10^. The mean eyebrow height for Malay male and Malay female in this study were 11.42 mm and 10.84 mm respectively. In comparison, the mean eyebrow height was 11.7 mm for male and 11.0 mm for female in Dharap & Reddys’ study^10^. One possible explanation for the similarities in result can be because of the further distance of eyebrow from upper eyelid lashes as compared to PTSH on primary gaze. Hence, there would be less eyebrow and eyelid movement during manual anthropometry measurement, resulting in more consistent measurements.

The mean measurement for upper eyelid crease height in our Malay subjects were higher compared to that reported by Dharap & Reddy^10^. The Malay male’s and female’s mean upper eyelid crease height in the Dharap & Reddy^10^ study were 6.1 mm and 6.5 mm respectively and coincided with the measurement reported by Preechawai^12^ on Thai-Malay, which was 6.6 mm. Upper eyelid crease height was measured with eye closed, thus the margin of errors from manual anthropometry would be less and may likely to arrive at the same result as 3D photogrammetry. However the result showed otherwise and possible explanation would be that, the Malay subjects from Dharap & Reddy^10^ and Preechawai^12^ were of Northern Peninsular Malaysia origin while the subjects of our study were more from the Central Peninsular Malaysia. It would be interesting from the anthropology point of view to investigate further on the possibility of eliciting differences in periorbital measurement between these Malay subgroups.

Wu *et al*.^11^ did an anthropometric study on 102 Chinese subjects. The eyebrow height in that study were divided according to gender and side of the eye as per current study. The Chinese male’s eyebrow height were 12.11 mm (right) and 12.53 (left), and the Chinese female’s eyebrow height were 12.22 mm (right) and 12.50 mm (left). All the figures presented by Wu *et al*.^11^ were higher than the eyebrow height reported in our study; this probably results from the usage of different definition of eyebrow height. According to Wu *et al*.^11^, the eyebrow height (known as upper eyelid height in his study) is the distance from orbital superius (Os) to palpebral superioris’ (Ps’). Os is the highest point on the lower border of eyebrow^14^. Ps’ is the intersection on the upper palpebral margin with vertical line through Os^11^. This measurement is different from that described by Dharap & Reddy^10^ whereby the eyebrow height was obtained at the midpupillary line, from the upper eyelid margin to the front row of mature eyebrow. Therefore the difference in definition of eyebrow height might have been the cause of the difference in eyebrow height reported in both studies.

Chen *et al*.^4^ did an anthropometric study on 260 Taiwanese Chinese women that was grouped according to age, and reported similar result in upper eyelid crease height with Sim *et al*.^3^. The age groups of our current interest are the 21–30 years old and 31–40 years old. For the age group of 21–30 years old, the pretarsal skin height was 2.4 mm and upper eyelid crease height was 6.0 mm. In the 31–40 years old group, the pretarsal skin height was 2.6 mm and upper eyelid crease height was 6.1 mm. Although these two studies were done using manual anthropometry, the result was close to our finding, in which Chinese female’s upper eyelid crease height was 5.89 mm. However, the upper eyelid crease height of 4.91 mm among Chinese subjects in this current study is higher than the 4.0 mm upper eyelid crease height reported by Preechawai^12^ in Thai-Malay.

Aljunid & Ngeow^13,19^ did a manual anthropometric study on 100 Malay subjects and 100 Chinese subjects in Malaysia. The result from their study was beneficial as it could be compared to our subjects whereby the intercanthal and interpupillary distances were similar between both studies. However, our study reported significant difference in interpupillary distance between the Malay and Chinese. As our study was done using 3D photogrammetry, any head movement that can happen as in manual measurement can be ruled out. Therefore, it can be concluded that Chinese had wider intercanthal distance than the Malays and the result is in agreement with that reported by Aljunid & Ngeow^20^. However, the Malays had wider interpupillary distance than the Chinese as a conclusion from our study alone. Our presumption regarding the results of intercanthal and interpupillary distances, which was based on the study by Al-Junid & Ngeow are reconfirmed by the current study, whereby Malay appears to have larger eyes that are located closer together.

Jayaratne *et al*.^15^ did a 3D photogrammetric study on Hong Kong Chinese subjects with similar samples size to the current study. However the intercanthal was larger compared to other previous results and to our current finding. The intercanthal distance reported by Jayaratne *et al*.^17^ was 40.01 mm for male and 38.27 mm for female. Thus, possible confounding factors such as epicanthal fold need be considered and re-examined in the future.

Lastly, our research also concluded that male of either ethnic groups tend to have a wider intercanthal and interpupillary distances than their female counterparts. This finding is in agreement to that reported by other researchers^7,13,21^.

One unique finding from this research was the significant prevalence of asymmetry in PTSH, EH and UECH obtained using 3D photogrammetry. This contradicts the conventional presumption that our periorbital soft tissues are symmetry. However any measurement with normal manual anthropometry would prove difficult especially in the periorbital region. That is the reason for the lack of information on the asymmetry of the right and left eyes’ periorbital tissue prior to the availability of photogrammetry.

By using 3D photogrammetry, we were able to detect minute differences between the right and left eye. It was found that there was *less* asymmetry in UECH and the distribution of right and left-sided dominant upper eyelid crease was almost equal in this study. However there were *more* asymmetry in PTSH and EH, with the left side being the dominant site. This can be explained by the fact that both the PTSH and EH were measured when the eyes were opened in primary gaze (which is subjected to muscle contraction of levator aponeurosis and frontalis muscle correspondingly). Therefore difference in the contraction of both muscles will manifest as the difference in heights measured. On the other hand, the UECH was measured with the eyelid closed, hence there was no active muscle contraction. Due to the significant difference in PTSH and EH between right and left eyes, it is suggested that aesthetic surgeons who are involved in blepharoplasty surgery perform pre-operative assessment using 3D-photogrammetry so that any asymmetry can be identified preoperatively and dealt with accordingly.

### Limitation of the Study

One limitation of this study was the challenge encountered when measuring the pretarsal skin height on eyes with more than one crease. The difficulty lies on making decision of the crease to be chosen as the point of measurement. In such a situation, we used the most superior and most dominant crease or fold as the point of reference.

Another limitation of this study was the fact that the upper eyelid crease line becomes vague once the eyes were closed especially in subjects with delicate upper eyelid crease. Therefore, zooming and searching for the crease on upper eyelid need to be done carefully when the eyes were closed in order to avoid wrong identification of wrinkles as upper eyelid crease. Nevertheless, our calibration study showed that we did not introduced unwanted error because of this faint appearance.

Lastly, we note that the epicanthal fold can be a confounding factor in intercanthal distance. Therefore, it is suggested that any future measurement of the intercanthal distance is done with the identification of epicanthal fold. Apart from that, any future eyebrow height measurement is suggested to be done with eye closed so that the measurement is less likely to be affected by the contraction of facial muscles.

## Conclusion

This study establishes that all Malay subjects have double eyelid but only 70.1% of Chinese subjects have double eyelid. The Malay has a higher pretarsal skin height, upper eyelid crease height and wider interpupillary distance compared to the Chinese. In contrast, the Chinese subjects have a higher eyebrow height and wider intercanthal distance. Lastly, there was significance differences in periorbital measurement between right and left eyes, which otherwise are commonly presumed to be symmetry.

## Methods

This is a descriptive and cross-sectional study that received ethical approval from the Medical Ethics Committee, Faculty of Dentistry, University of Malaya (Reference Number: DF OS1404/0012(P)). This study protocol adhered strictly to the principles of the Declaration of Helsinki. It was carried out at the Department of Oral and Maxillofacial Clinical Sciences, Faculty of Dentistry, University of Malaya between May 2014 and April 2015. Subjects with normal facial appearance but attending the Oral Surgery clinic for routine dental procedures such as dental extractions and/or third molar surgeries were invited to participate in this study.

We have excluded subjects with history of periorbital trauma or surgery, congenital craniofacial abnormalities, had orbital or eyelid tumors or suffering from disease that affects the orbit and/or its surrounding soft tissue, such as Graves’ disease, myasthenia gravis, cerebral palsy and strabismus. In addition, those with a history of periorbital swelling or scarring, wearing contact lens, having eye tape or glue or had underwent eyebrow shaving were also excluded.

**Figure 1:**
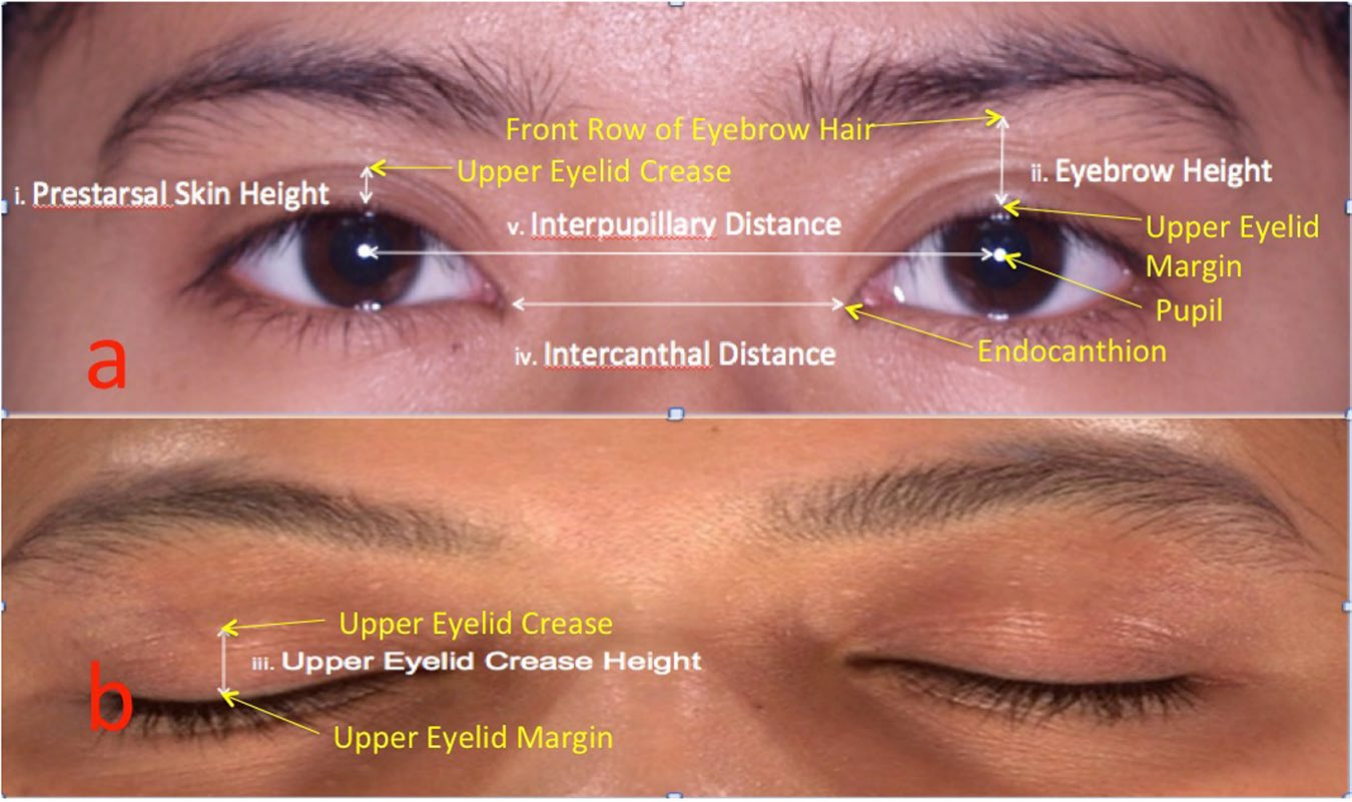
(a) Measurement with eyelid opened. (b) Measurement with eyelid closed.

Informed written consent including possible publication of the subject’s image online and in printed media was obtained from all persons recruited. They were required to cover their hair with a swimming cap (male) or a headscarf (female) with only the facial region including the frontal region and ear exposed. This was done to prevent inconsistency in measurement due to distraction from differing hair volume^22^. The subject was seated with his/her Frankfurt horizontal plane parallel to the floor and 3D images of the face were captured using VECTRA-3D 360 5 pod-mounted photosystem (Canfield Scientific Inc., Fairfield, NJ, USA). The images were captured in two eyelid positions, which were the primary gaze (for the measurement of PTSH, EH, ICD and IPD) (Fig. 1a) and the closed eye position (for the measurement of UECH) (Fig. 1b).

Colour texture images will be displayed on the computer screen, immediately followed by monochrome surface images. Green and red elements will begin to appear on the images for the system to build the 3D model. Capture process occurred in less than 2 milliseconds and the automatic 3D processing took less than a minute. This will result the 3D image being stored as a file on the computer and displayed on the screen^23^.

The acquired 3D images, available in raw format, were exported from the 3D camera, and transferred to a standard desktop computer that was installed with the corresponding imaging software, 3D Mirror Software (Canfield Scientific Inc., Fairfield, NJ, USA) provided by the manufacturer. The processed 3D images were analyzed by a single investigator (TYL) who identified 5 landmarks according to current standard and accepted definition^10,14^. In summary, he marked the midpupil, the upper eyelid crease and upper eye lid margin, the front row of eyebrow hair and the endocanthion accordingly with a cursor attached to a computer mouse. To reduce errors, midpupillary line for measuring PTSH and EH, and mid-distance for measuring UECH were marked on the digital images to produce consistent localisation of the landmarks. Thereafter, five linear measurements (*see description in the next paragraph*) that were located between these landmarks were generated by the software and the results were recorded accordingly in an Excel spreadsheet. They were the:

i. pretarsal skin height (PTSH)
ii. eyebrow height (EH)
iii. upper eyelid crease height (UECH)
iv. intercanthal distance (ICD)
v. interpupillary distance (IPD)

The percentage of errors of measurements was calculated using the Bland & Altmann formula.

### Ethical approval

This study was approved by Medical Ethics Committee, Faculty of Dentistry, University of Malaya (Reference Number: DF OS1404/0012(P)).

## Periorbital Measurements

The pretarsal skin height (PTSH) is the distance between the upper eyelid margin (eyelid lash) and the upper eyelid crease, observed at the midpupillary line with the eyes in primary gaze (Fig. 1a). The eyebrow height (EH) is the distance between the upper eyelid margin (eyelid lash) and the front row of mature eyebrow hairs of the inferior eyebrow margins, observed at the midpupillary line also with the eyes in primary gaze (Fig. 1a). The upper eyelid crease height (UECH) is the distance between the mid-horizontal distance of the upper eyelid margin (eyelid lash) and the mid-horizontal distance of upper eyelid crease when the eyes were closed (Fig. 1b)^10^. The intercanthal distance (ICD) is defined as the distance between the right endocanthion and left endocanthion which is sustained by the median canthal ligament (Fig. 1a) that connects the tarsal plates and palpebral structures to the median orbital structures^24^. The interpupillary distance (IPD) is the distance between the right midpupil and the left midpupil (Fig. 1a).

Sample size calculation based on the results reported by Farkas^14^ suggested that at least 35 subjects were required in each ethnic group to obtain significant results. Two calibration tests were performed for this study; one was to ensure that 3D photogrammetry was accurate, and another one was to ensure intra-examiner reliability. Metzler *et al*.^22^ had earlier determined the validity of the 3D VECTRA photogrammetric surface imaging system for cranio-maxillofacial anthropometric measurements but we needed to confirm this ourselves to ensure that the results obtained were accurate^25^. This was done by comparing the results for the 5 parameters described above, obtained from 10 subjects using the 3D photogrammetry with those obtained using manual calipers. In manual anthropometry, the landmarks on the actual subjects were measured using a pair of Mitutoyo digital caliper. Although currently 3D photographic imaging have been claimed to become new gold standard in facial anthropometry, one cannot deny the fact that manual measurement is still the well tried and tested method of craniofacial anthropometry^20^. Once accuracy and validity was confirmed, the defined measurements were collected for all subjects recruited. The investigator then took a break before repeating the measurements for the purpose of intra-examiner calibration, at least 2 weeks from the first exercise. This process was done on 10% of randomly selected subjects. This will ensure that all data presented in this study was reliable, consistent and reproducible. Statistical analysis was performed using Statistical Package for Social Science (SPSS) software program (Version 22.0; SPSS Inc., Chicago, IL, USA) using intra-class coefficient (ICC) test for both purposes.

Other data analysis was done according to the specific objectives of the study. Pearson Chi-Square test was used to investigate the association between the prevalence of double eyelid with ethnic. This test is chosen because the prevalence of double eyelid is a continuous variable but ethnic is a dichotomous variable. Kolmogorov–Smirnov test was used to check for distribution normality of the dependent variables. It was noted that the distribution of the EH, ICD and IPD were normal, hence independent *t*-test was performed to compare for significant difference of these parameters. However, paired sample *t*-test was the test recommended to analyse for statistical difference of EH between the right and left eye.

The data for PTSH and UECH were skewed in their distribution, as confirmed using Kolmogorov-Smirnov test. Thus, the non-parametric test of Mann-Whitney was performed to evaluate for significant difference of these parameters. Following the statistician’s recommendation, Wilcoxon test was used to determine for statistical difference of PTSH and UECH between right and left eye. The level of significant was set at α = 5% (*p* < 0.05).
